# Comparative Study of Hydrothermal and Sonochemical Preparation of Cu and Ag Codoped ZnO/Graphene Nanocomposites for Enhanced Catalytic and Inactivation of Pathogens

**DOI:** 10.1002/cphc.202500256

**Published:** 2026-02-15

**Authors:** Jarvin Mariadhas, Vinodhkumar Ganesan, Sudhan Narayanan, Sarojini Jeeva Panchu, Hendrik C. Swart, Nelson Y. Dzade, Savariroyan Stephen Rajkumar Inbanathan

**Affiliations:** ^1^ Post Graduate and Research Department of Physics The American College Madurai Tamil Nadu India; ^2^ Post Graduate and Research Department of Physics Raja Doraisingam Government Arts College Sivagangai Tamil Nadu India; ^3^ Post Graduate and Research Department of Chemistry Thiagarajar College Madurai Tamil Nadu India; ^4^ Department of Physics University of the Free State Bloemfontein South Africa; ^5^ Department of Energy and Mineral Engineering Pennsylvania State University University Park, State College Pennsylvania USA

**Keywords:** antibacterial activity, graphene nanocomposites, hydrothermal and two‐dimensional materials, photocatalytic activity, Zn‐O‐C interactions

## Abstract

Developing high‐performing photocatalysts and composites generate synergetic effects in modern photocatalysis. Zinc oxide (ZnO) is a promising photocatalyst; however, its wide bandgap and high charge carrier recombination rate significantly limit its visible‐light activity and overall photocatalytic efficiency. To overcome these challenges, this study focuses on the synthesis of Cu–Ag codoped ZnO/graphene nanocomposites using both hydrothermal and sonochemical methods, aiming to regulate the interfacial interaction and enhance charge separation. The hydrothermally synthesized (CAZ/Gr)_H_ composite exhibited a lower bandgap, improved carrier transfer efficiency, and stronger Zn–O–C interfacial bonding compared to the sonochemically prepared (CAZ/Gr)_S_ sample. Density functional theory (DFT) calculations confirmed the reduced work function and enhanced electron mobility in the hydrothermal system. Under natural sunlight, the (CAZ/Gr)_H_ composite demonstrated superior photocatalytic degradation of organic dyes and excellent antibacterial activity against *E. coli* and *S. aureus*. These findings highlight the effectiveness of interface‐regulated, green‐synthesized ZnO‐based nanocomposites in addressing the fundamental limitations of traditional ZnO photocatalysts.

## Graphical abstract

Cu–Ag codoped ZnO/graphene nanocomposites were synthesized via hydrothermal and sonochemical methods to enhance interfacial charge transfer and visible‐light activity. Strong Zn–O–C bonding, reduced bandgap, and improved electron mobility enabled superior photocatalytic dye degradation and antibacterial performance.

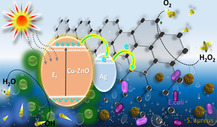

## 1 Introduction

Manufacturing of a wide range of consumer items, natural, and synthetic dyes have been widely used in the food, paper, textile, pharmaceutical, cosmetic, and printing industries. Water is used extensively throughout the dyeing process and then improperly released into the environment as an aqueous effluent. Malachite green and methyl orange are the most often used dyes and pigments among the over 10,000 different types utilized in different sectors [1, 2, 3]. When these dye‐infused products are utilized, they are released into the environment and have an impact on the ecosystem's flora and fauna. Discarded effluents increase the chemical oxygen demand (COD), limit visibility, and slow down photosynthesis, all of which have an impact on both aquatic and terrestrial life [4, 5, 6]. According to the natural selection principle, bacteria have developed mechanisms to resist antibiotics ever since they were first introduced and used to cure diseases. Antibiotic overuse in recent years has resulted in several issues related to harmful effects, particularly the emergence of resistance strains. Since most harmful bacteria are resistant to antibiotics, their efficiency has diminished. Gram positive Staphylococcus aureus (S. aureus) and gram‐negative Escherichia coli (E. coli), which have a widespread distribution in living beings, soil, water, and plants, are two of the most significant sources of infection in people, especially nosocomial infections [7, 8, 9, 10]. The use of metal and oxide nanoparticles, hetrostructure, and nanocomposites is one of the most promising, novel strategies against bacterial resistance and catalysts for the degradation of dye that has been proposed [11, 12, 13].

Among various photocatalysts, ZnO is a widely used UV light catalyst due to its larger bandgap of 3.3 eV, physical and chemically stable in environment, affordability, accessibility, and high oxidative capacity [14, 15, 16, 17]. These excellent properties make ZnO a suitable material for various applications such as laser diodes, solar cells, and catalytic reactions [18, 19, 20, 21, 22, 23, 24]. The main advantage of ZnO is that it is nontoxic, hence is commonly used for biological applications. However, the major drawback of the ZnO is its wide bandgap, photo‐corrosion, and high recombination rate between electrons and holes, which reduces the efficiency of its photocatalytic activity. The bandgap of ZnO can be reduced via the addition of an extra impurity, which creates new energy levels and gives rise to visible light absorption [25, 26, 27, 28, 29]. Depending on the doping material, the bandgap and electronic properties of the host material can be tuned. New acceptor energy levels are created near the conduction band when the ZnO is doped with metal ions while new donor energy levels are formed above the valence band ZnO doped with nonmetals [30, 31]. Metal doping also greatly influences the surface property of the nanocomposite particularly on the surface hydroxyl of the photocatalyst [32]. Among the various explored transition metals, Cu and Ag are demonstrated to be suitable dopants for narrowing the bandgap of ZnO. The ionic radii of Cu is similar to Zn, and it is nontoxic and less expensive and has a higher electronegativity than Zn, which makes it a perfect dopant for the nanocomposites (NCs). The incorporation of a noble metal like Ag can produce more photogenerated charge carriers under sunlight illumination due to the localized surface plasma resonance effect and processes effective dye removal as well as antibacterial activity [33, 34, 35, 36].

Due to the rapid recombination of the photogenerated charge carriers, which reduces the photocatalytic performance of ZnO photocatalysts, the coupling of ZnO with other materials to form heterostructures can overcome the fast recombination drawback [37, 38, 39, 40]. Generally, carbon‐based materials are attractive materials to couple with ZnO owing to their high stability, high surface area, and high conductivity [41, 42, 43, 44]. For, instance, Kalisamy et al. [45] demonstrated that the creation of a heterojunction between ZnO and S‐doped g‐C_3_N_4_ nanoparticles to form a Z‐scheme photocatalyst, providing effective separation of electrons and holes in the opposite direction, and it is advantageous for the absorption of visible light for photocatalytic dye degradation. Graphene also belongs to the carbon family, and it possesses a novel sp^2^ hybridized carbon layer structure in two dimensions. It is a zero bandgap with elevated electrical conductivity for storing and transporting electrons through *π*‐*π* interaction. Also, graphene has a high theoretical specific surface area of 2600 m^2^g^−1^ due to its one‐atom thickness [46]. The high surface area provides more active sites for the catalytic reactions and also helps to suppress electron–hole recombination [47]. The environmentally high stability nature of graphene also makes it a preferable partner material for ZnO to achieve enhanced photocatalytic activity. Concerning this, Pham Van Tuan et al. [48] reported that by adding an appropriate amount of rGO (ZnO/rGO 10%), a significant improvement in the dye removal activity through the efficient separation of photo‐induced electron and hole was obtained. Li Liu et al. [49] prepared nitrogen‐doped graphene‐ZnO using a sol–gel method. Their findings reveal of the addition of GO (2 wt% of NG‐ZnO) enhanced the catalytic activity via narrowing of the bandgap as well as diminishing the charge carriers from recombination. Electro‐spun synthesized nano‐fibered graphene‐ZnO has been reported to exhibit better catalytic activity than pure ZnO. The enhanced activity has been attributed to the addition of a suitable amount of graphene, which greatly suppresses photoinduced electrons and holes recombination, as reported by Seongpil An et al. [50]. On the antibacterial front, recent reviews and experimental studies highlight that ZnO nanoparticles kill bacteria through a combination of mechanisms: (i) generation of ROS (e.g., •OH, O_2_^‐^) under irradiation or in dark via defect sites, (ii) release of Zn^2+^ ions that disrupt intracellular metabolic activity, and (iii) direct physical interaction with and disruption of the bacterial cell membrane/wall [51, 52]. In graphene‐ or dopant‐modified ZnO nanocomposites, the enhanced interfacial contact further increases ROS production and facilitates electron transfer to bacterial membranes, thereby boosting antibacterial efficiency. By integrating interface‐regulated hydrothermal synthesis and dopant/graphene engineering, the present study addresses both the visible‐light‐utilization barrier and the antibacterial mechanism gap of ZnO‐based nanocomposites.

Several approaches have been developed in recent years, including sol–gel thermal oxidation, sonochemical, combustion, and rapid precipitation methods for the preparation of ZnO nanoparticles [53, 54]. With the help of these techniques, many morphologies for ZnO have been produced, including nanowire, nanorod, nanoneedle, nanoflower, and nanoparticles. The chemical and biological activities of nanomaterials are influenced by their morphology [55, 56]. The green synthesis approach is one of the most affordable, straightforward, less agglomeration, and efficient methods among all the suggested methods for the preparation of metal oxide nanoparticles [57, 58, 59, 60]. Catechins, a flavonol type of polyphenols, are abundant in fresh tea leaves and make up around 30% of the dry leaf weight. Flavonoids and their glycosides, chlorogenic acid, gallic acid, coumarylquinic acid, and theogallin are some of the additional polyphenols that are found in leaves. Green leaf polyphenols are often protected from oxidation by not fermenting green tea. In terms of chemical composition, green tea is fairly similar to fresh tea leaves, with the exception of a few enzymatically catalyzed alterations that happen very quickly after plucking. Tea extract serves as both a capping agent and a reducing agent in the production of metal oxide NPs [61]. Potassium is present in considerable amounts in tea extract, ranging from 92 to 151 mg/L, along with other metals including sodium (35 to 69 mg/L), calcium (1.9 to 3.5 mg/L), fluoride (0.8 to 2.0 mg/L), aluminum (1.0 to 2.2 mg/L), manganese (0.52–1.9 mg/L), and iron (0.020 to 0.128 mg/L). Polyphenols are present in tea extract and function as antioxidants. Epicatechin gallate (ECG) (203–471 mg/L), epigallocatechin gallate (EGCG) (117–442 mg/L), epicatechin (EC) (25–81 mg/L), and epigallocatechin (EGC) (16.9–150 mg/L) are the four flavonoid groups that make up this phenolic group. The main group in epigallocatechin (EGC) in the reduction process is ‐OH [62].

In the present study, we have synthesized a Cu and Ag incorporated ZnO‐embedded graphene composite (CAZ/Gr) using two different green and sustainable approaches: sonication and hydrothermal method. Cu and Ag metals were incorporated into the ZnO NCs and deposited over the surface of the graphene layer in a single step. The prepared nanocomposite photocatalysts were examined by various characterization techniques, and their effectiveness towards malachite green (MG) and methyl orange (MO) dye degradation and antibacterial activities was tested. The (CAZ/Gr)_H_ nanocomposite delivered excellent degradation efficiency towards MG dye and efficient antibacterial activity against S‐aureus and E. coli than the (CAZ/Gr)_S_ and CAZ NCs.

## 2 Experimental Section

### 2.1 Materials and Methods

For the synthesis of (CAZ/Gr) nanocomposite, zinc acetate dihydrate (Zn (CH_3_COO)_2_· ·2H_2_O), silver nitrate (AgNO_3_), and copper nitrate trihydrate (Cu(NO_3_)_2_) were used as a starting materials (analytical grade from a Merck (India)_. Pure graphene was purchased from KNVS incorporation (Nagpur, India). The *Camellia sinensis bags* (green tea bags) were purchased from a private shop tea vendor (Madurai, India). Double‐deionized water was used as a solvent for all experiments.

### 2.2 Cu‐Ag‐ZnO/Graphene Prepared by Sonication Method ((CAZ/Gr)_S_)

Tea extracts were prepared based on our previously reported work [63]. One mmol of Zn(CH_3_COO)_2_·2H_2_O was added to the 160 mL of tea extract sonicate (frequency 2450 MHz, 700 W) for 30 min; then, 0.1 mmol of AgNO_3_ was added and sonicated for 15 min, followed by the addition of 0.1 mmol of Cu(NO_3_)_2_. ·3H_2_O. After 1 h of sonication, 0.32 g of graphene was added to the as prepared mixture to sonicate well for the time period of 2 hr at room temperature.

### 2.3 Cu‐Ag‐ZnO/Graphene Prepared by Hydrothermal Method ((CAZ/Gr)_H_)

**FIGURE 1:**
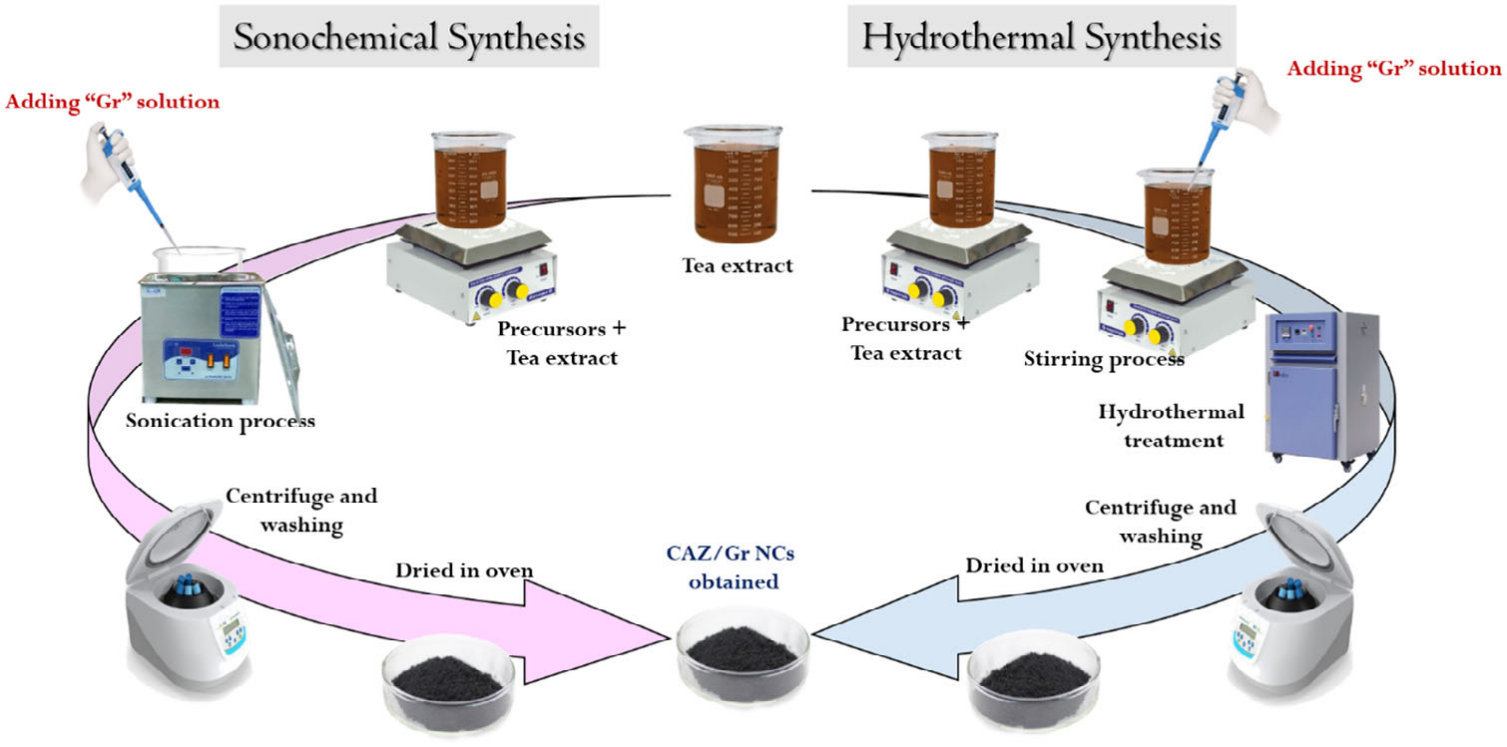
(CAZ/Gr)_S_ and (CAZ/Gr)_H_ NC preparation procedure.

One mmol of Zn(CH_3_COO)_2_·2H_2_O was added to the 160 mL of tea extract and stirred for 30 min. Once the Zn was mixed well with the tea extract, we added 0.1 mmol AgNO_3_ to the solution and stirred it for 15 min, followed by the addition of 0.1 mmol of Cu(NO_3_)_2_·3H_2_O. After a 1 h stirring process, 0.32 g of graphene was added to the above mixture and stirred well. Then, the stainless‐steel autoclave was filled with the mixture and hydrothermally treated for 2 h at 180ºC. In the above two synthesis methods, the final products were centrifuged and cleaned by using ethanol and DI water 3 times to remove the impurities and dried in a hot air oven at 60º C overnight. The schematic of the CAZ/Gr nanocomposite preparation is shown in Figure 1. The Cu‐Ag‐ZnO (CAZ) nanoparticles were synthesized by the coprecipitation method using tea extract as a capping agent.

### 2.4 Characterization of NCs

Analysis of the structure and phase of the synthesized NCs was carried out using an Ultima III max (Rigaku) X‐ray powder diffractometer (XRPD) using Cu K*α* radiation (*λ *= 1.54 Å, 40 Kv, 100 mA) at a scan rate of 2ºmin^−1^. Fourier transforms spectrophotometer (JASCO FT/IR‐4600) was used to analyze the molecular properties of the NCs. A high‐resolution field emission scanning electron microscope (HR‐TEM) equipped with an energy dispersive X‐ray spectrometer (EDS) was used to examine the morphology and chemical composition of the prepared NCs (SIGMA HV – CARL ZEISS with Bruker Quantax 200 – Z10 EDS Detector). A Renishaw Invia Raman microprobe was used to record Raman spectra from 200 to 2000 cm^−1^ using a 514.5 nm argon ion laser. The surface area, pore volume, and pore diameter of the NCs were determined by N_2_ adsorption using the Brunauer–Emmett‐Teller (BET) and Barret‐Joyner‐Halenda methods. UV absorption and concentration of dye in the photocatalytic experiment were carried out by JASCO V‐730 spectrophotometer. Room temperature photoluminescence (PL) spectra of the synthesized NCs were recorded using a spectrofluorometer (JASCO FP‐ 8300) at an excitation wavelength of 350 nm.

### 2.5 Photocatalytic Experiment

The decolourization of dyes using the prepared photocatalysts was evaluated under sunlight. Methyl orange (MO) and malachite green (MG) dye were used as model pollutants at the concentration of 5 ppm (5 mg/L). The experiments were conducted during consecutive sunny days on June 08–12, 2023, between 10:00 am and 1:00 pm at Madurai, with GPS coordination of 9.9759 °N, 78.1393 °E. The solution temperature was maintained between 28 and 32ºC. MG and MO dyes have their characteristic absorption peaks at 621 and 465 nm. Both the dyes were under sunlight irradiation without photocatalyst for more than 150 min, and no noticeable changes in the color of dyes or absorbance had been observed (photolysis process). A significant amount of photocatalyst (0.012 g) was mixed into the 150 mL of 5 ppm MG and MO dyes. Before being kept under sunlight, the adsorption–desorption equilibrium was reached after magnetic stirring for 30 min in the dark. The 5 mL of aliquots were collected only when the equilibrium was reached and referred to as the concentration at zero time. Once the solution was placed under the sunlight, 5 mL of aliquots was collected at appropriate interval times. The aliquots that were collected undergo centrifugation and the degradation of MG and MO dyes was examined by UV–Visible absorption spectroscopy with distilled water as the reference medium (wavelength range of 200–800 nm). *C*_t_ is the concentration at a reaction time “t,” and *C*_0_ is the initial concentration of the MG and MO dyes. The percentage of degradation of dye solutions was calculated from the equation [63]

(1) $$\mathrm{Degradation}\mathrm{efficiency}(\%)=\frac{C_{0}-C_{t}}{C_{0}}\times 100$$

### 2.6 Dye Degradation Kinetics

The degradation rate of MG and MO dyes was identified by using the most commonly used pseudo‐first and second‐order reactions. The first‐order kinetic linear form model is expressed as follows [64, 65]

(2) $$\ln\;\left(C_{0}/C_{t}\right)=k_{1}t$$

The second‐order kinetic linear form model is expressed as follows

(3) $$1/C_{t}-1/C_{0}=k_{2}t$$

where *C*_0_ and *C*_t_ denotes the concentrations of MG and MO dyes, respectively, at time *t*_0_ and given time t, respectively. The rate constants for first‐ and second‐order kinetics are *k*_1_ (min^−1^) and *k*_2_ (min^−1^). For the first‐order kinetic plot of the graph between ln (*C*_0_/*C*_t_) vs time, the slope of the curve gives the value of the rate constant (*k*_1_). For the second‐order kinetics plot, the graph between 1/*C*_t_‐1/*C*_0_ vs the time slope of the curve gives the value of rate constants (*k*_1_&*k*_2_). Linear regression analysis gives the value of correlation coefficient value (*r* ^2^).

### 2.7 Antibacterial Tests

The antibacterial activity test was practically examined by the disc diffusion method [66, 67]. Escherichia coli (MTCC 732) and Staphylococcus aureus (MTCC 3160) were the bacterial strains used in the biological experiments. The Institute of Microbial Technology (IMTECH), Chandigarh, India, was where the bacterial strains were bought. Thirty milliliters of NA (Nutrient Agar) medium was poured into the Petri plates. The test organism was spread on a solidified agar plate and allowed to dry for 10 min. A sterile cotton swab dipped into a standardized microbial culture is used to spread the microorganisms evenly on the entire surface of the Nutrient agar plates. Using sterile forceps, the sterile filter papers (6 mm diameter) containing 50, 100, and 150 μL of samples (CAZ, (CAZ/Gr)_S_, (CAZ/Gr)_H_) and a readily available antibiotic chloramphenicol and fluconazole discs were used as a standard. A control plate with 30 μL (DMSO) was also plated. The plates were incubated at 37 ºC for 24 h. Each sample was tested in triplicate. The mean millimeter diameter of the zone of inhibition surrounding the disc was used as the basis for calculating the antibacterial potential of test substances. The zones of inhibition of the studied microorganisms by the substances were measured on a millimeter scale.

### 2.8 Computational Details

The density functional theory (DFT) calculations were performed using Projector Augmented Wave (PAW) pseudo‐potential method [68], as implemented in the Vienna Ab initio Simulation Package (VASP) [69, 70]. The Perdew–Burke–Ernzerhof (PBE) function was used to approximate the exchange–correlation [68, 71, 72]. An energy cut‐off of 600 eV was used for the wave function, and the convergence criteria for the residual Hellmann–Feynman forces were set to 0.001 eV/Å. The Hubbard DFT + U approach was used to provide an accurate treatment of the electron correlation in the localized Zn‐*d* orbitals, ensuring accurate determination of the electronic bandgap of the investigated materials. Based on previous results theoretical results [73, 74], the effective U value for Zn, Cu, and Cu were set to 10 eV, whereas that of O was set to 7 eV, predicting the bulk bandgap at 3.24 eV for pristine ZnO. From the optimized bulk ZnO material, the (10$\bar{1}$0) surface, which is often predicted as one of the most stable surfaces for ZnO, was created and used for the construction of the CAZ (10$\bar{1}$0) surface and CAZ (10$\bar{1}$0)/Gr composite. The CAZ (10$\bar{1}$0) surface model was built by substitutionally doping one Cu and Ag ions at the top surface of Zn ion sites, where they are energetically most favored. The CAZ (10$\bar{1}$0)/Gr composite was constructed with a (5 × 5) supercell of the CAZ (10$\bar{1}$0) surface and a (5 × 5) graphene supercell. The CAZ (10$\bar{1}$0) surface is comprised of 10 atomic layers with periodic boundary conditions, and a 20 Å vacuum was added in the z‐direction to prevent interactions between periodic images. A Monkhorst–Pack (76) k‐point mesh of 5 × 5 × 1was used to sample the Brillouin zone of the ZnO (10$\bar{1}$0), CAZ (10$\bar{1}$0), and CAZ (10$\bar{1}$0)/Gr composite, respectively.

## 3 Results and Discussions

### 3.1 Structural, Morphological, and Compositional Analyses

**FIGURE 2:**
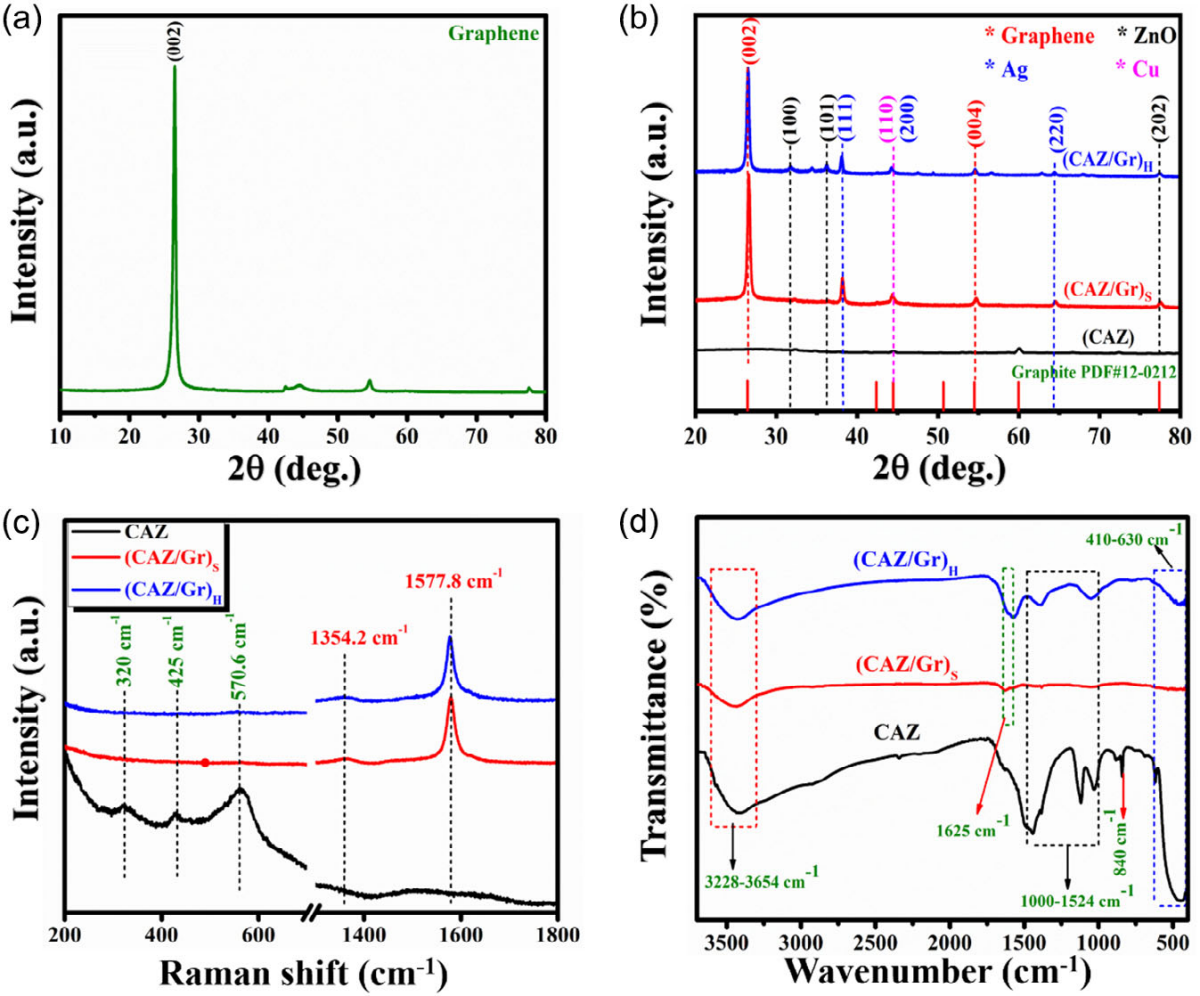
(a) XRD pattern of graphene, (b) CAZ, (CAZ/Gr)_S_, and (CAZ/Gr)_H_ NCs, (c) Raman, and (d) FTIR of CAZ, (CAZ/Gr)_S_, and (CAZ/Gr)_H_ NCs.

The structure and crystallinity of the prepared photocatalysts were characterized through XRPD. Figure 2b shows the XRPD patterns of CAZ, (CAZ/Gr)_S_, and (CAZ/Gr)_H_. The diffraction peaks at the position (2*θ*) of 31.98°, 36.11°, and 77.3° correspond to the *hkl* planes (100), (101), and (202), respectively, confirming the the hexagonal phase wurtzite ZnO (JCPDS No: 80–0075) with high crystallinity [75, 76]. The peaks located at 38.15°, 44.29°, and 64.52° could be indexed to be cubic phase silver (JCPDS No: 87‐0597) [77, 78, 79]. The peak at the position of 44.4° is due to the incorporation of Cu. These results demonstrate the successful modification of ZnO surface with Cu and Ag particles. In the (CAZ/Gr)_S_ and(CAZ/Gr)_H_ NCs, the most dominant sharp peak at the region of 26.4° can be attributed to the (002) lattice plane of graphene with the corresponding to the *d*‐spacing of 3.4 Å (JCPDS No: 12‐0212) [80, 81] as shown in Figure 2a. These findings clearly show that CAZ nanoparticles are decorated on the surface of the graphene sheet, which is consistent with TEM results. The bare CAZ, (CAZ/Gr)_S_, (CAZ/Gr)_H_ and NCs show all the diffraction patterns for Cu, Ag, and ZnO showing the low‐intensity peaks; this is due to the high level of dispersion and low concentration of Cu, Ag, and ZnO as well as high crystalline nature of graphene sheets.

Figure 2c depicts the Raman spectra of the bare CAZ, (CAZ/Gr)_S_, and (CAZ/Gr)_H_ NCs. The bare CAZ exhibits three dominant peak positions at 320, 425, and 570.6 cm^−1^. The peaks at 320 and 425 cm^−1^ are due to the second‐order Raman spectrum emerging from the zone‐boundary phonon and nonpolar optical phonon E_2_ vibration mode of the ZnO hexagonal structure. The dominant peak at 570.6 cm^−1^ corresponds to the E1 LO (longitudinal optical) mode of the ZnO structure [82, 83, 84]. For (CAZ/Gr)_S_ and (CAZ/Gr)_H_ NCs, the small intensity peak corresponds to CAZ, resulting from the small doping amount of Cu, Ag, and ZnO. On the other hand, the two high‐intensity peaks in (CAZ/Gr)_S_ and (CAZ/Gr)_H_ NCs located at 1354.2 and 1577.8 cm^−1^ can be assigned to the D and G bands of graphene. The D band arises due to the defects created by the oxygenated functional groups on the surface of graphene. The prominent peak of 1577.8 cm^−1^ clearly shows the high‐quality sp^2^ hybridized carbon atoms present in the NCs [48, 50]. The small intensity of the D band also indicates the graphene is of good quality (lack of defects). The intensity ratio of (*I*_D_/*I*_G_) of the D and G band in (CAZ/Gr)_S_ and (CAZ/Gr)_H_ nanocomposite were determined 0.3544 and 0.4551, respectively. Basically, the (*I*_D_/*I*_G_) ratio increases with increasing defects with oxygenated functional groups. The (*I*_D_/*I*_G_) ratio for (CAZ/Gr)_S_ is smaller than that of (CAZ/Gr)_H_ [48], presented in Table 1.

**TABLE 1:** Raman intensity and peak positions for the CAZ, (CAZ/Gr)_S_, and (CAZ/Gr)_H_ NCs.

Samples	Raman shift, cm^−1^					
	Cu	Ag	ZnO	Gr	Intensity, a.u.	*I*_D_/*I*_G_
CAZ	539.67 619.54	1364 1524.76	320 425 570.6	—	—	—
(CAZ/Gr)_S_	—	—	320.54 424.76 570.8	1368.2 1577.8	3386 1200	0.3544
(CAZ/Gr)_H_	—	—	320.54 424.76 570.8	1354.2 1577.8	6908 15195	0.4551

Actually, the basal plane of the graphene contains a small amount of highly stable in‐plane oxygenated functional groups (epoxy, carbonyl, and carboxylic) evidently proved by (*I*_D_/*I*_G_) ratio; these functional groups are directly connected to the defect sites in the CAZ NPs possibly via the Zn‐O–C bond (chemisorption) to facilitate the hybrid formation due to the high pressure as well as high temperature produced during the hydrothermal process [85, 86]. This effective bond greatly influences the catalytic behavior. At the same time, during the sonication process, the CAZ NPs loosely combined over the surface of the graphene sheet through weak van der Waals forces (physisorbed), which is greatly supported by our TEM results (discussed in the following section). This clearly shows that the hydrothermal method is an effective method for the synthesis of CAZ/Gr NCs compared to the sonication method.

The various functional groups present on the surface of the as‐prepared photocatalysts and their vibrational modes were analyzed using FTIR spectroscopy. The FTIR spectrum of prepared NCs is shown in Figure 2c. The absorption band observed from 3228–3654 cm^−1^ was ascribed to the stretching vibration mode of water molecules (H‐O‐H) present in the surface of the samples and the N‐H stretching mode of polyphenols such as hydroxyflavones and catechins [87]. In addition, the peak in the range between 1000 and 1524 cm^−1^ related to the C‐O‐C, O‐H/N‐H, and C = C stretches of polyphenols groups present in the tea extract [88]. The peak at the range of 1590 cm^−1^ in the samples (CAZ/Gr)_S_ and (CAZ/Gr)_H_ may indicate the remaining sp^2^ bond (C = C) in graphene [48, 77]. The broad vibration band at 1625 cm^−1^ was observed in the (CAZ/Gr)_H_; it is possibly due to the Zn‐O–C bond formation during the hydrothermal process. The peak observed at 840 cm^−1^ could be attributed to the alkene group (C–H) out of plane bending. The peaks from 410–630 cm^−1^ are due to the metal oxide vibration [M‐O] vibration modes of Zn‐O‐Zn, Zn‐O‐Ag, and Zn‐O‐Cu [39, 43, 89]. These interactions suggest the formation of (CAZ/Gr) nanocomposite. Note that in (CAZ/Gr)_H_ NCs, all the absorption peaks are broadened and highly intense as compared to that of bare and (CAZ/Gr)_S_ NCs, which indicates the effective interaction created between the Ag, Cu, and ZnO with graphene during the hydrothermal synthesis through various bond formation.

The structural morphology of the CAZ, (CAZ/Gr)_S_, and (CAZ/Gr)_H_ NCs is shown in Figure S1. The FESEM image of the CAZ NPs shows the nanoplates with a small granular structure of particle size of about ~102 ±  60 nm (Figure S1a,b). The (CAZ/Gr)_S_ clearly shows the small granular size of ~37±20 nm CAZ NPs nonhomogenous distributed over the surface of graphene. This clearly shows that the NPs are not effectively interact with thick graphene sheets during the sonication process (Figure S1c,d). The tea extract may hinder the growth of NPs in large sizes during the NCs formation. The (CAZ/Gr)_H_ NCs show a particle size of 55 ± 30 nm; the graphene sheets are visible, with fine CAZ NPs loaded over the sheets. Graphene also may hinder the NPs from agglomeration. There is no discernible interlayer gap between the thin layer of graphene and the CAZ NPs created during the hydrothermal process, indicating strong adhesion produced between the two materials through the effective bond of Zn‐O‐C. CAZ and graphene have such a strong connection that enhances the transport of electrons during photocatalysis. Particularly, the large interfacial area provided by the graphene's thin sheets makes it easier for dye adsorption and surface‐dependent redox processes to occur (Figure S1e,f) [48, 49]. The in‐depth morphological details about the interaction are discussed in the following section. The presence of elements are confirmed through the EDS analysis (Fig S2).

**FIGURE 3:**
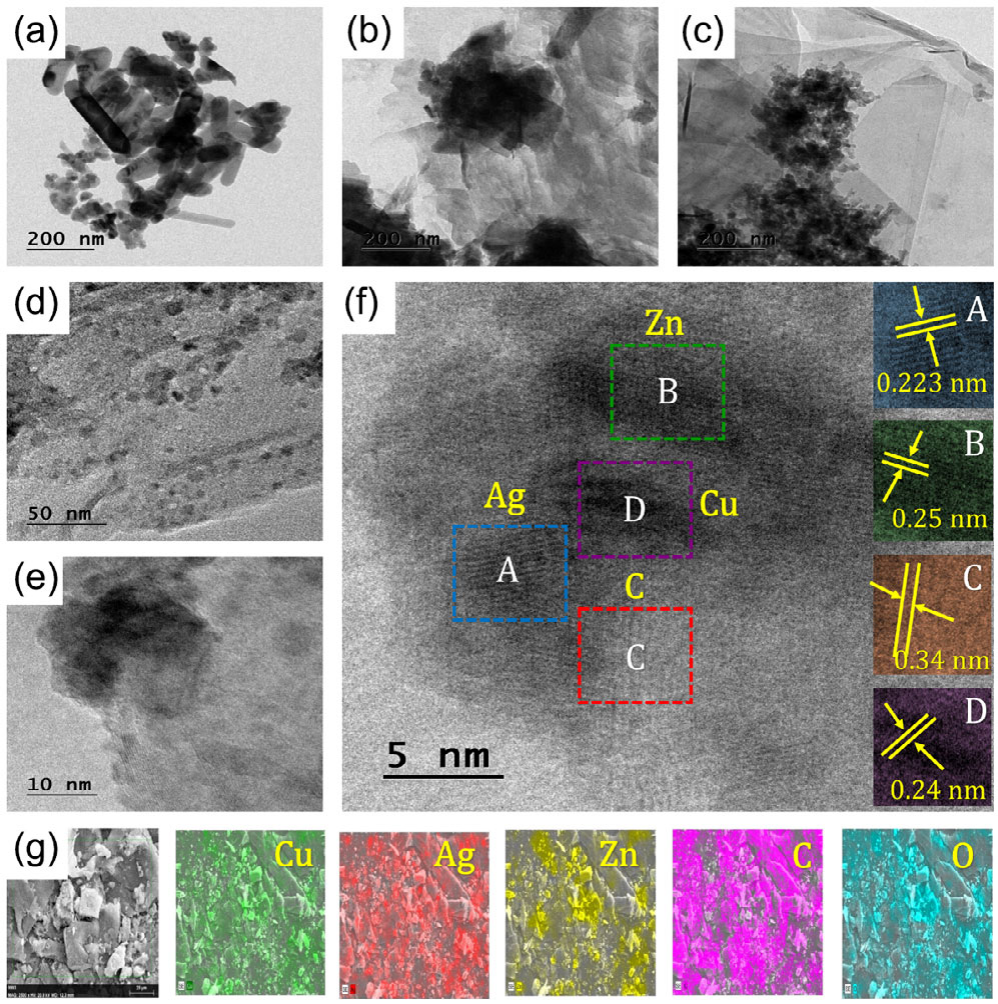
Low magnification TEM images of (a) Bare CAZ, (b) (CAZ/Gr)_S_, and (c) (CAZ/Gr)_H_. (d,e) Two different magnifications (50 and 10 nm) and (f) HR‐TEM images of (CAZ/Gr)_H_ (the insets of A, B, C, and D depict the corresponding lattice fringe images of Cu, Ag, Zn, and graphene respectively). (g) Selected area of mapping and corresponding EDX mapping images of the (CAZ/Gr)_H_ NCs.

Figure 3 presents TEM images of NPs of CAZ (Figure 3a), (CAZ/Gr)_S_ (Figure 3b), and (CAZ/Gr)_H_ NCs (Figure 3c). The shape of the bare CAZ NPs are rod as well as the spherical shape like structure (ZnO—rod shape; Cu and Ag—spherical shaped). (CAZ/Gr)_S_ NCs exhibited the hazy wrinkled thick sheets of graphene with the CAZ NPs aggregated over the thick sheets, so the individual particles look like a cluster. But hydrothermally synthesized NCs depict the single‐layered graphene sheets with the partial distribution of CAZ NPs Figure 3d. Reduced particle size in the range of ~17 nm CAZ NPs loaded over the high surface graphene sheets; reduced particle size NPs give more reactive active sites for the reaction [32, 37]. The partial distribution is due to the high concentration of graphene compared to the CAZ NPs. At the same time, the partial distribution shows better interaction between the NPs and the graphene compared to the cluster (prepared by the sonication method). Moreover, the high‐resolution TEM images give a detailed view of the interaction between the NPs and the graphene sheet. It is clearly seen that the well‐defined interlunar *d*‐spacing of 0.24, 0,223, 0.25, and 0.34 nm were corresponding to the Cu, Ag, Zn, and graphene, respectively Figure 3f [75, 77, 81]. Most importantly, the Cu and Ag‐ ZnO (metal–metal oxide) Schottky potential junction was clearly seen in Figure S3. The Schottky junction act as the potential barrier to prevent or mitigate the photogenerated charge carriers from recombination. The enhanced lifetime of the photogenerated charge carriers is evidently proved by the PL studies also. The electrically interconnected particles of Cu and Ag both could act as the trapping center for the electrons; this will enhance the photocatalytic activity through the effective production of ROS (reactive oxidative species). The distribution of Cu, Ag, Zn, C, and O in the (CAZ/Gr)_H_ NCs is clearly seen in Figure 2g.

TEM, HR‐TEM, and mapping images of CAZ and (CAZ/Gr)_S_ NCs are presented in Figure S4,S5, respectively. The CAZ NPs were present in spherical shape along with lattice fringes of Cu, Ag, and Zn are clearly discernible. (CAZ/Gr)_S_ NCs show the uneven distribution of NPs over the graphene sheets. The edges of the sonicated graphene layers are thicker compared to the hydrothermally prepared NCs. It is noted that as compared to (CAZ/Gr)_H_, the lattice spacing of the (CAZ/Gr)_S_ NCs is marginally increased, due to the strain created in the structure due to the ultrasonication waves. These results again confirm that the synthesis method played a vital role in the morphology of the (CAZ/Gr) NCs. Highly dispersed thin layers of graphene sheets with reduced particle size NPs are more favorable for the degradation of organic pollutants [42, 43].

### 3.2 Surface Area and Composition Analyses

N_2_ adsorption–desorption isotherms and pore size distribution measurements of the CAZ, (CAZ/Gr)_S_, and **(**CAZ/Gr)_H_ composites were calculated using the BET (Btunauer–Emmett–Teller) method. As shown in Figure S6a–c, the results show a type IV isotherm, which depicts the NCs contains number of mesopores. The specific surface areas of bare CAZ, (CAZ/Gr)S, and (CAZ/Gr)H nanocomposites were approximately 6.54, 9.98, and 33.76 m^2^ g^−1^, respectively. The corresponding average pore diameters and total pore volumes were 7.5 nm and 0.0361 cm^3^ g^−1^ for CAZ, 10.3 nm and 0.0540 cm^3^ g^−1^ for (CAZ/Gr)S, and 21.8 nm and 0.180 cm^3^ g^−1^ for (CAZ/Gr)H, as obtained from the BJH pore‐size distribution (Figure S6d). The surface area of the (CAZ/Gr)_S_ and(CAZ/Gr)_H_ NCs is almost 1.5 and 5.5 times larger compared to the bare CAZ composite. The larger surface area, pore volume, and pore size for the (CAZ/Gr)_H_ could facilitate more active sites for the reaction [88, 90], offer an effective transit route for the photogenerated charge carriers to the interior void area, and provide good absorption surface to the dye molecules, which suggests that they may be advantageous for photocatalytic activity [91, 92]. These germane properties suggest that the (CAZ/Gr)_H_ composite would be a highly efficient photocatalyst for dye degradation and antibacterial activity.

**FIGURE 4:**
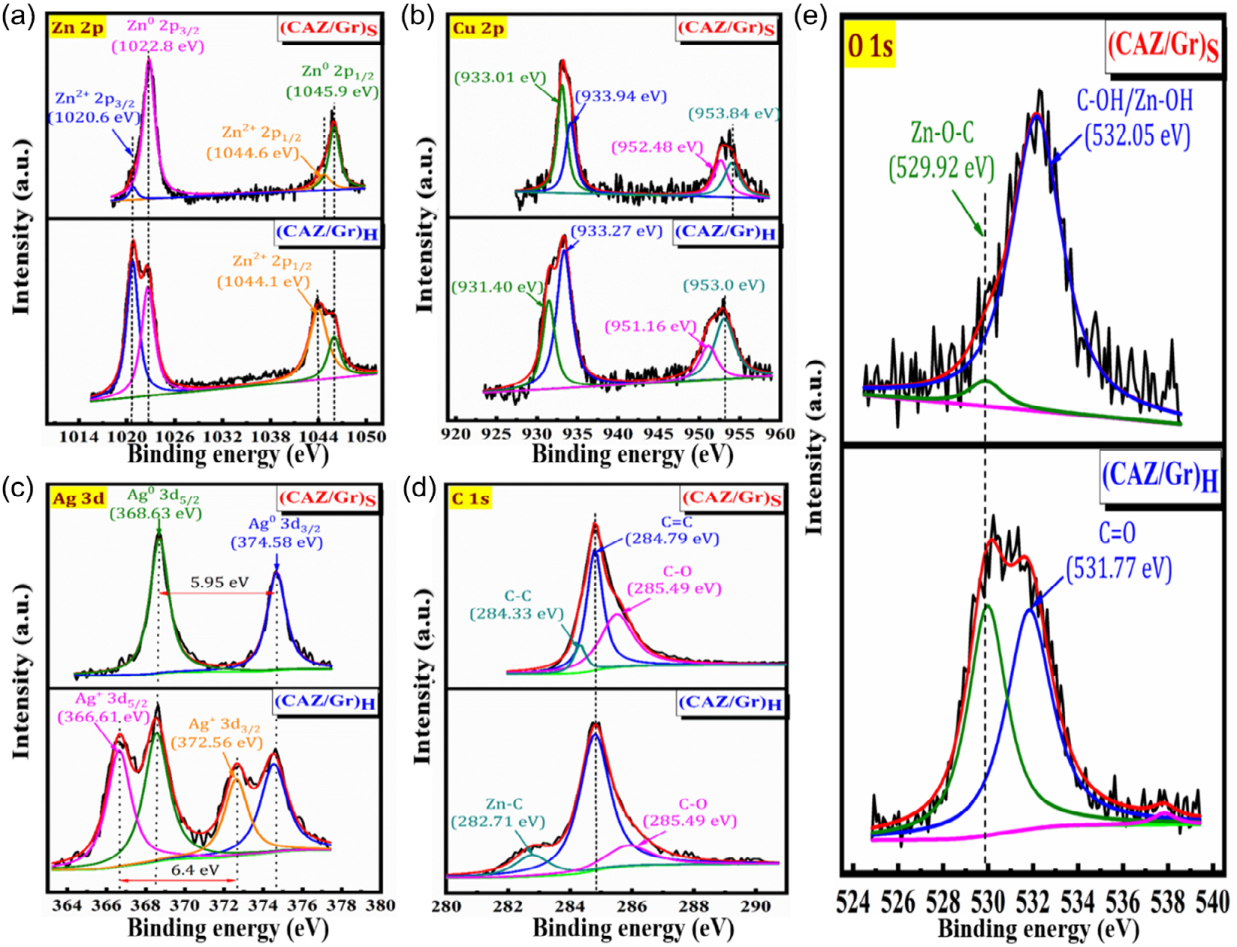
XPS patterns of (a) Zn 2p, (b) Cu 2p, (c) Ag 3d, (d) C 1s, and (e) O 1s spectrum of prepared (CAZ/Gr)_S_ and (CAZ/Gr)_H_ NCs.

The XPS spectrum of the CAZ, (CAZ/Gr)_S_, and (CAZ/Gr)_H_ NCs are displayed in Figure 4 and Figure S7. In Figure 4a, the two major peaks located at the binding energies of 1022.8 and 1045.9 eV were assigned to the Zn 2p_3/2_ and Zn 2p_1/2_ of Zn. The deconvolution of Zn 2p spectra shows two new peaks at two different positions at 1020.6 and 1044. 6 eV associated with the Zn 2p_3/2_ and Zn 2p_1/2_, respectively, revealing Zn to be in +2 oxidation state [93, 94]. The intensity of the +2 oxidation peak is higher for (CAZ/Gr)_H_ NCs compared to the (CAZ/Gr)_S_ NCS, which might be due to the oxidation process occurring over the surface of the sample when it is exposed to air. The Cu 2p spectrum (Figure 4b) exhibits peaks at 931.4 and 953.0 eV, which corresponds to the divalent state (Cu^2+^) of Cu 2p_3/2_ and Cu 2p_1/2_. The Cu 2p deconvoluted spectrum shows two peaks at 931.4 and 951.2 eV, respectively, exhibiting Cu 2p in the metallic state (Cu^0^). This result indicates that the prepared NCs contained both mixed states of Cu NPs [95, 96]. Further, the Ag 3d spectra (Figure 4c) show it had two sets of peaks of 368.6 and 374.6 eV corresponding to Ag 3d_5/2_ and Ag 3d_3/2_, respectively, and the spacing between the two peaks was 5.95 eV, which confirms that the presence of Ag NPs in the Ag^0^ metallic state. (CAZ/Gr)_H_ NC portrayed the extra new peaks at 372.6 and 366.6 eV may reveal the existence of Ag^+^; the energy difference between these two peaks was about 6.4 eV, which is greatly supported by the already reported data [97, 98, 99]. The C 1s deconvolution spectrum (Figure 4d) revealed three sets of C‐binding. Among these, the peak at the location of 284.8 eV and 285.5 eV correspond to the C = C and C‐O groups. Another small peak at 284.3 eV for (CAZ/Gr)_S_ samples depicts the C–C, and the 282.7 eV for (CAZ/Gr)_H_ sample is possible for the metallic Zn directly bonded with carbon (Zn‐C) [100, 101]. The O 1s (Figure 4e) spectrum of (CAZ/Gr)_S_ and (CAZ/Gr)_H_ was deconvoluted in two components at the energies of 529.9 eV (Zn‐O‐C) and 532.05 eV (C‐OH/Zn‐OH) for (CAZ/Gr)_S_ and 529.9 eV (Zn‐O‐C) and 531.8 eV (C = O) for (CAZ/Gr)_H_ NC. The intensity of the peak at the binding energy located at 529.9 eV (Zn‐O‐C) gives very useful information about the interaction between the metal oxides with the Gr sheets [100, 102, 103]. The higher intensity for the (CAZ/Gr)_H_ NC reveals stronger chemisorbed interaction (Zn‐O‐C) created between the metal oxides with the Gr sheets; also, the Gr is successfully integrated with the CAZ NPs during the hydrothermal process. Also, the observed negative shift in the Zn 2p and O 1s binding energies indicates electron transfer from graphene and dopant atoms (Cu and Ag) toward ZnO, which enhances charge carrier separation and suppresses recombination. This interfacial charge redistribution is further supported by DFT results showing a lower work function and improved electron mobility for the hydrothermal composite. Therefore, the superior photocatalytic and antibacterial performance of (CAZ/Gr)_H_ is primarily attributed to this efficient interfacial charge transfer through Zn–O–C bonds, and its serves as the key factor facilitating visible‐light‐driven redox reactions. The peak width of Zn (Figure 4a) gives some more details about the interaction between the materials; higher FWHM values, 3.7 eV for the (CAZ/Gr)_H_ NCs and 2.1 eV for the (CAZ/Gr)_S_ NCs, have been observed, suggesting the emergence of a Zn‐O–C interaction, as it has been seen in the C1s spectra [101].

### 3.3 Optoelectronic and Photoluminescence Property Analyses

The optical property is one of the most important factors that dictate photocatalytic activity. As per literature studies, the bandgap of the pristine ZnO is large (~3.3 eV); it can absorb only in the UV‐light source [16, 17]. The doping with Cu and Ag is expected to decrease the bandgap an increase the absorption range towards the visible region. Figure S8 a shows the UV–reflectance spectrum of CAZ, (CAZ/Gr)_S_, and (CAZ/Gr)_H_ samples. The CAZ exhibits reflectance towards the visible region with a reflecting edge occurring at 407 nm. The incorporation of Cu and Ag into ZnO leads to an increase the visible light absorption. After making composite with graphene, the (CAZ/Gr)_S_ and (CAZ/Gr)_H_ depicts the peak around 245 nm (*π*‐π* transition) due to electronic conjugation in the graphene [104, 105]. (CAZ/Gr)_S_ and (CAZ/Gr)_H_ NCs can absorb more light in the visible region (400–800 nm) as compared to the CAZ samples. This characteristic clearly shows that the introduction of Cu, Ag, and graphene into the ZnO samples effectively broadens their light absorption as well as diminished the recombination rate through the creation of a Schottky barrier with the CAZ NPs. Figure S8b shows plots of Kubelka–Munk remission ((F(R) h*ν*)^2^ versus photon energy (h*ν*)) functions corresponding to CAZ, (CAZ/Gr)_S_, and (CAZ/Gr)_H_ NCs. The bandgap value obtained from these graphs is 3.2 eV for CAZ, 1.58 eV for (CAZ/Gr)_S_, and 1.46 eV for (CAZ/Gr)_H_ nanocomposite.

**FIGURE 5:**
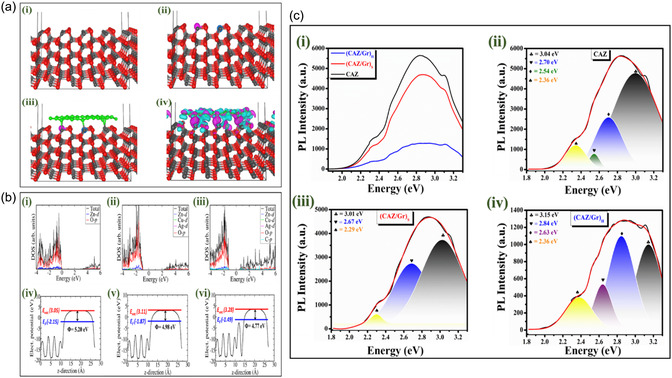
(a) Optimized structures of the (i) ZnO (10$\bar{1}$0), (ii) CAZ (10$\bar{1}$0), and (iii) CAZ (10$\bar{1}$0)/Gr composite and (iv) differential charge density iso‐surfaces, where the green and purple contours indicate the electron density increase and decrease by 0.05 e/Å^3^, respectively. Atomic colors: Zn = dark gray, O = red, Cu = light blue, Ag = pink, and C = green. (b) The partial density of states (i–iii) and electrostatic potentials (iv–vi) of ZnO (10$\bar{1}$0), CAZ (10$\bar{1}$0), and CAZ (10$\bar{1}$0)/Gr composite, respectively. *E*_vac_, *E*_F_, and *Φ* in eV correspond to the vacuum level, Fermi level, and work function. (c) PL intensity graph of (i) combined (ii) CAZ, (iii) (CAZ/Gr)_S_, and (iv) (CAZ/Gr)_H._.

From first‐principles DFT calculations, the structures and electronic properties (Figure 5a) of the pristine ZnO (10$\bar{1}$0), Cu‐Ag‐doped ZnO (10$\bar{1}$0) surface (CAZ), and the CAZ/Gr composites were systematically characterized. The CAZ/Gr composite is stabilized via the formation of Zn‐O‐C bonds (Figure 5aiii), confirmed by differential charge density analyses, which reveal electron density accumulation in the Zn‐O‐C bonding regions (Figure 5iv). The interactions within the (CAZ/Gr)_H_ composite gave rise to the tuning of the electronic structure: shifting the Fermi level towards to the edge of the conduction band and reducing the bandgap relative to the pristine ZnO and CAZ (Figure 5b. The bandgap of the pristine ZnO (10$\bar{1}$0) surface is calculated at 3.19 eV compared to 2.3 eV for the CAZ (10$\bar{1}$0) and 1.67 for CAZ(10$\bar{1}$0)/Gr composite. Due to the synergistic electronic interactions between graphene and CAZ and CAZ (10$\bar{1}$0)/surface, we also observed a decrease in the work function of the CAZ(10$\bar{1}$0)/Gr composite (4.77 eV) compared to the CAZ(10$\bar{1}$0) surface (4.98 eV) and the pristine ZnO (10$\bar{1}$0) surface (5.20 eV). The reduction in the work function and bandgap of the CAZ (10$\bar{1}$0)/Gr composite is expected to facilitate the rapid transport of electrons from the valence to the conduction band to participate in photocatalytic reactions.

Photoluminescence spectroscopy (PL) is a simple and widely used technique for characterizing or exploring the destiny of photogenerated electron–hole pairs in a semiconductor. Before diffusing to the CAZ surface, the photogenerated electron can recombine with holes [43], leading to a release of energy that results in fluorescence. Based on this characteristic of the CAZ NPs, the interaction between CAZ and graphene can be analyzed by measuring the fluorescence decay of the nanocomposite. PL emission spectra of the as‐synthesized NCs were recorded between 370 and 625 nm, with an excitation at a wavelength of 350 nm, and are plotted in Figure 5c. All three samples’ PL spectra displayed multiple emission bands. Curve fitting establishes each PL spectrum's peak energy. The three samples exhibit broad distinct peaks from UV to visible light ranges. The peak at ~3.1 eV (~390 nm) (♣) arises from the ZnO‐free excitonic transition between the conduction and valence band. And the broad emission peak from 2.6–2.8 eV (400–500 nm), which includes the three different emissions of blue (2.81 eV, “♦”), green (2.58 eV, “♥”), and yellow (2.31 eV, “♠”) emission, is generally nominated as DLE (Deep Level Emission). DLE arises due to the various intrinsic defects in ZnO structure, namely oxygen vacancy (V_0_), interstitial oxygen (O_i_), oxygen antisite, zinc vacancy (Zn_0_), interstitial zinc (Zn_i_), and zinc antisite [106]. Higher intensity PL peak indicates a higher rate of recombination, and lower intensity peak corresponds to lower recombination of photogenerated charge carriers [43, 88]. Among the three samples, the visible PL intensity of CAZ is increased by 4.5 fold, and (CAZ/Gr)_S_ is increased by 3.5 fold compared to that of (CAZ/Gr)_H_ NCs. CAZ NPs show the highest intensity peak, indicating a higher rate of recombination. (CAZ/Gr)_S_ and (CAZ/Gr)_H_ NCs exhibit a lower intensity compared to CAZ NPs. This is due to the addition of graphene acting as a potential barrier for the excited electron, which significantly helps to suppress the recombination rate. At the same time, for (CAZ/Gr) NCs, the defect emission band is greatly mitigated due to the healing of surface defects after the addition of graphene; defect sites can significantly affect the catalytic behavior. These results are consistent with previous investigations, which show that ZnO decorated with graphene can greatly influence in electron–hole recombination rates [50, 107, 108].

### 3.4 Photocatalytic Degradation of MG and MO Dyes Over CAZ, (CAZ/Gr)_S_, *and* (CAZ/Gr)_H_ NCs

**FIGURE 6:**
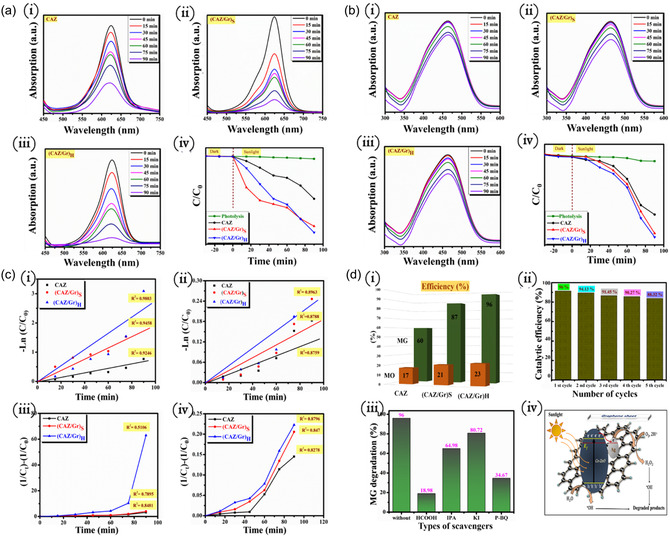
Photocatalytic dye degradation graphs for (a) MG dye (b) MO dyes in the presence of catalyst (i) CAZ, (ii) (CAZ/Gr)_S_, (iii) (CAZ/Gr)_H_ NCs, and (iv) degradation efficiency graph. (c) CAZ, (CAZ/Gr)_S_, and (CAZ/Gr)_H_ NC (i) pseudo‐first‐ and (iii) second‐order kinetic graphs of MG dye and (ii) pseudo‐first‐ and (iv) second‐order kinetic graphs of MO dyes. (d) (i) Percentage of MO and MG dye degradation graph of CAZ, (CAZ/Gr)_S_, and (CAZ/Gr)_H_. (ii) Reusability test of (CAZ/Gr)_H_ NCs for the degradation of MG dye under simulated sunlight irradiation. (iii) Scavenger's effect on the photocatalytic degradation of MG of (CAZ/Gr)_H_ NCs. Initial conditions: concentration of MG dye = 5 ppm, catalyst dosage = 0.012 g, the volume of dye solution = 150 mL, and the number of cycles = 5, and (iv) Photocatalytic dye degradation mechanism of CAZ/Gr NCs.

Malachite green (MG) and methyl orange (MO) pollutant degradation was carried out on the CAZ, (CAZ/Gr)_S_, and (CAZ/Gr)_H_ NCs in order to measure and compare their photocatalytic activities. The photocatalytic degradation profile of the two dyes, photocatalyzed by CAZ, (CAZ/Gr)_S_, and (CAZ/Gr)_H_ NCs are shown in Figure 6a, b. During the photolysis process, no color changes were noticed in the MG and MO dye. On the other hand, the dye removal efficiency increased to 60 % for CAZ NPs. This is due to the creation of an extra energy band (Cu^2+^) created near the conduction band and Schottky barrier (Ag) in the CAZ NPs. This will effectively increase the sunlight absorption and diminish the electrons and hole recombination rate. After the hybridization of CAZ NPs to the graphene heterojunction of CAZ/Gr formed, the degradation efficiency is enhanced, portrayed in Figure 6a (ii and iii). The hydrothermally synthesized NCs depict a vast degradation efficiency than the sonochemically synthesized NCs, possibly due to the effective chemisorbed Zn‐O–C bond formed within the (CAZ/Gr)_H_ than the loosely attached NPs over the surface of CZA NPs during the sonication process. For the MG dye, CAZ shows lesser efficiency (60 %) than the (CAZ/Gr)_S_ (87%) and (CAZ/Gr)_H_ (96%) NCs within 90 min. This clearly demonstrate that the integration of graphene into the CAZ NPs enhances the catalytic activity through effective chemisorbed bond creation. The hydrothermally prepared CAZ/Gr NCs show better activity than the sonochemical prepared ones, suggesting that the preparation method also plays a crucial role in the photocatalytic activity. The degradation efficiency is found to be in the order of CAZ < (CAZ/Gr)_S_< (CAZ/Gr)_H_. It was found that the type and nature of the dye also influences its degradation process, as the MG dye shows a better degradation efficiency than the MO dye (Figure 6b). This can be attributed to the electrostatic attraction force created between the catalyst and the dye, i.e., MO dye is anionic in nature, whereas the MG dye is cationic in nature. So, the electrostatic repulsion and attraction force takes place between the dye molecules and the photocatalyst [109, 110, 111]. Due to the electrostatic repulsive force, the MO dye shows lesser efficiency than the MG dye.

Figure 6c shows the kinetics graphs of the MG and MO dyes. From the kinetic studies, the first‐ and second‐order kinetic values for CAZ are 0.8759 and 0.8273, for (CAZ/Gr)_S_ are 0.8788 and 0.847, and for (CAZ/Gr)_H_ are 0.8963 and 0.8796, respectively. This shows that MO dye follows second‐order kinetics for all three NCs. Similarly, for the MG dye, CAZ and (CAZ/Gr)_S_ also follow the second‐order kinetics, while the (CAZ/Gr)_H_ follows the first‐order kinetics. (CAZ/Gr)_H_ shows an r^2^ value of 0.9803 and 0.5106 for first‐ and second‐order kinetics, respectively, indicating that the first‐order model is the most suitable method for the degradation of MG dye. Figure 6di depicts the degradation percentage efficiency of MO and MG dye over the CAZ, (CAZ/Gr)_S_, and (CAZ/Gr)_H_ NCs. The stability of the highly efficient (CAZ/Gr)_H_ NCs was evaluated by testing them against the MG dye in five consecutive cycles (Figure 6dii); the degradation efficiency decreased only ~7% compared to the initial cycle. This decrease may be due to the catalyst loss during the centrifugation process or intermediates of MG which get absorbed on the catalyst surface. Table 2 shows the kinetics and correlation values of MG and MO dyes.

**TABLE 2:** Kinetics and correlation values of MG and MO dyes.

Samples	Methyl orange (MO)				Malachite green (MG)			
	First‐order model		Second‐order model		First‐order model		Second‐order model	
	Rate constant, min^−1^	*r* ^2^	Rate constant, min^−1^	*r* ^2^	Rate constant, min^−1^	*r* ^2^	Rate constant, min^−1^	*r* ^2^
CAZ	0.0021	0.8759	0.0017	0.8278	0.0077	0.9246	0.0308	0.8481
CAZ/Gr)_S_	0.0027	0.8788	0.0022	0.847	0.0204	0.9458	0.031	0.7895
(CAZ/Gr)_H_	0.0029	0.8963	0.0024	0.8796	0.0298	0.9803	0.3368	0.5106

In order to find out the effective role of ROS during the catalytic degradation, the scavenger experiment was carried out using different scavengers like IPA (^•^OH), P‐BQ (^•^ $O_{2}‐$), HCOOH (e^‐^), and KI (h^+^) in the presence of (CAZ/Gr)_H_ NCs under the illumination of sunlight irradiation, and the acquired results are depicted in Figure 6diii. The catalytic efficiency of MG dye decreased from 96 %, 80.72 %, 64.98 %, 34.67 %, and 18.98 % without scavengers, in the presence KI, IPA, P‐BQ, and HCOOH. No noticeable degradation in efficiency was observed in the presence of IPA and KI, but the catalytic efficiency effectively diminished in the presence of P‐BQ and HCOOH scavenger. Based on the above discussion, ^•^ $O_{2}‐$ and e^‐^ play a major role in the degradation of MG dye. The photocatalytic process and mechanism of semiconducting CAZ, (CAZ/Gr)_S_, and (CAZ/Gr)_H_ under sunlight irradiation is given as follows: (i) photoexcitation, (ii) generation of charge carriers, (iii) migration of electron, and (iv) separation of carriers, as shown in Figure 6div. When the light energy incident on the NCs is equivalent to or greater than the bandgap energy, the electrons are excited from the valence band to the conduction band, leaving a hole behind. The excited electrons will be transferred to the defect state of the extra energy band Cu^2+^ and trapped by the Ag and accumulate over the surface. This will effectively prevent the recombination rate.

Generally, the Ag dopant produce more electron under visible light due to the LSPR (localized surface plasma resonance) effect. These electrons migrate to the graphene surface via surface defects due to the low work function value of graphene compared to ZnO and Ag. The bare CAZ NPs show lower activity compared to CAZ/Gr composite. The incorporation of graphene into the composite enhances the photocatalytic activity due to the extraordinary properties like (i) high conductivity nature due to its 2D planar structure, (ii) high surface area gives sufficient active sites for the reaction, (iii) graphene suppresses the electron and hole recombination rate, and (iv) graphene prevent the photocatalyst in photo‐corrosion. The reduction and oxidation process occurs at the surface of graphene and the ZnO valence band through the effective production of ROS. Electrons on the surface of graphene will react with oxygen molecules to form superoxide anions (^•^ $O_{2}‐$). At the same time, holes in the ZnO valence band reacts with water to form ^•^OH radicals. These reactive species break the MG dye chromophores into small constituent particles (CO_2_ and H_2_O and other intermediate products). Table 3 shows that the photocatalytic activity on the degradation of various color dyes is compared with hydrothermally synthesized (CAZ/Gr)_H_ photocatalysts for the degradation of malachite green.

**TABLE 3:** Shows that the photocatalytic activity on degradation of various color dyes is compared with hydrothermally synthesized (CAZ/Gr)_H_ photocatalysts for the degradation of malachite green.

S. no.	Catalyst	Degrading dye	Synthesis method	Degradation time, min	Efficiency, %	References
1	ZnO	MB	Green synthesis	100	91	[24]
2	Ag, Cu‐ZnO	MB	Coprecipitation	180	92	[36]
3	Carbon doped ZnO	MB	Urea‐assisted method	120	90	[40]
4	rGO‐ZnO	AO7	UV‐assisted reduction method	300	97	[75]
5	Co‐ZnO	MO	Coprecipitation	120	54	[111]
6	La‐ZnO	MB	Wet chemical	100	83	[112]
7	Ag‐ZnO	MB	Green synthesis	100	96	[113]
8	ZnO‐GO	Cv	Chemical method	80	95	[114]
9	ZnO/GO	MO	Hydrothermal	120	95	[115]
10	Sodium alginate/ZnO/GO	Cv	Copolymerization	300	94	[116]
11	Cu, Ag‐ZnO/Gr	MG	Hydrothermal	90	96	This study

### 3.5 Analysis of the Antibacterial Activity of CAZ, (CAZ/Gr)_S_, and (CAZ/Gr)_H_ NCs Over the Two Nonidentical Bacteria

**FIGURE 7:**
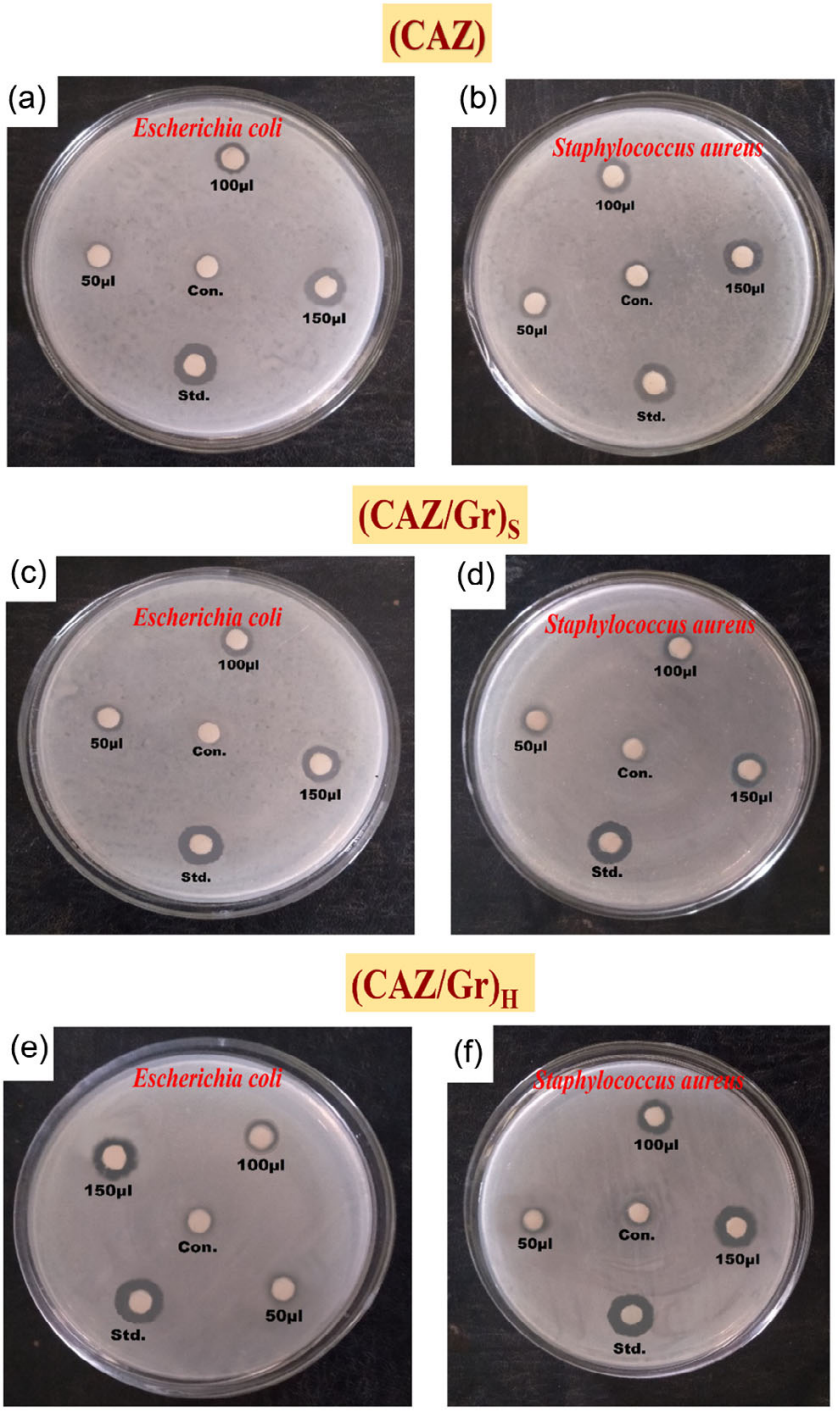
Antibacterial activity of CAZ, (CAZ/Gr)_S_ and (CAZ/Gr)_H._.

The antibacterial activity of the prepared three NCs using three different concentrations (50, 100, and 150 μL) is shown in Figure 7. Depending on the concentration of the NCs used, the antibacterial activity also differs for both bacteria. As the concentration of the NCs was increased, the zone of inhibition also increased. The (CAZ/Gr)_S_ and (CAZ/Gr)_H_ NCs show better activity than the CAZ, which can be attributed to the coupling of graphene with the CAZ. All three NCs show better activity towards gram‐negative than the gram‐positive bacteria. Reactive oxidative species (ROS) such as oxygen, OH radicals, and O^2^‐ are produced by the Cu, Ag, ZnO, and graphene during the reaction [9, 117, 118]. These radicals cannot penetrate the cell membrane of the negative bacteria. In the case of the positive bacteria, Cu, Ag, ZnO, and graphene produced free radicals that can enter into the cell wall and bind with the DNA molecules and make structural changes in the cell membrane by creating stress, resulting in cell death [10, 119]. It also assists Ag, Cu, ZnO, and graphene in evenly distributing over the NCs. The antibacterial activity for both bacteria with the different NCs is found to be in the order of CAZ < (CAZ/Gr)_S_< (CAZ/Gr)_H_, as shown in Table 4.

**TABLE 4:** Antibacterial activity report of CAZ, (CAZ/Gr)_S_, and (CAZ/Gr)_H_ NCs.

Samples	*Bacterial strain*	Sample concentration				
		50 µL Zone of inhibition, mm	100 µL Zone of inhibition, mm	150 µL Zone of inhibition, mm	Std., 30 µL	Control, 30 µL
CAZ	*Escherichia coli*	2.85 ± 0.19	5.50 ± 0.38	9.00 ± 0.63	12.45 ± 0.87	0.10 ± 0.007
	*Staphylococcus aureus*	2.50 ± 0.17	5.30 ± 0.37	8.75 ± 0.61	11.70 ± 0.82	0.05 ± 0.003
(CAZ/Gr)_S_	*Escherichia coli*	3.00 ± 0.21	5.70 ± 0.39	9.10 ± 0.63	12.50 ± 0.86	0.10 ± 0.007
	*Staphylococcus aureus*	2.80 ± 0.19	5.50 ± 0.38	8.95 ± 0.62	11.75 ± 0.82	0.05 ± 0.003
(CAZ/Gr)_H_	*Escherichia coli*	3.15 ± 0.22	6.10 ± 0.42	9.85 ± 0.68	12.55 ± 0.87	0.10 ± 0.007
	*Staphylococcus aureus*	3.05 ± 0.21	5.90 ± 0.41	9.55 ± 0.66	11.80 ± 0.82	0.05 ± 0.003

## 4 Conclusions

In summary, we report the successful synthesis of CAZ, (CAZ/Gr)_S_, and (CAZ/Gr)_H_ NCs through a sonication and hydrothermal approach using tea extract as a reducing agent. The incorporation of Cu and Ag into the ZnO is shown to reduce the bandgap of the nanoparticles towards the visible region and increase the light absorption ability. It was demonstrated that the loading of CAZ nanoparticles over the graphene sheet effectively inhibited the recombination of charge carriers. The (CAZ/Gr)_S_ and (CAZ/Gr)_H_ NCs also provided maximum effective surfaces for reaction and high photostability towards achieving enhanced photocatalytic and antibacterial activity. Complementary first‐principles DFT calculations demonstrated that the formation of Zn‐O‐C chemisorbed bond in the (CAZ/Gr)_H_ composite results in bandgap and work function reduction, which promotes efficient separation and transfer of electrons‐hole pairs compared to isolated ZnO (10$\bar{1}$0) and CAZ (10$\bar{1}$0) surfaces towards achieving improved photocatalytic activity. These results demonstrate that the prepared (CAZ/Gr)_H_ NCs are promising photocatalysts for environmental remediation and antibacterial applications.

## Supporting Information

Additional supporting information can be found online in the Supporting Information section. FESEM/HRTEM images, EDS, elemental mapping, BET surface area and pore diameter graph, CAZ XPS graph, UV and Tauc's plot. **Supporting Fig. S1:** FESEM image of (a) CAZ NPs, (c) (CAZ/Gr)S, and (e)(CAZ/Gr)H, and the size distribution of the (b) CAZ NPs, (d) (CAZ/Gr)S, and (f)(CAZ/Gr)H based on the FESEM image. **Supporting Fig. S2:** EDX images of (a) CAZ NPs, (c) (CAZ/Gr)S, and (e) (CAZ/Gr)H. **Supporting Fig. S3:** Schottky potential barrier in (CAZ/Gr)H NCs. **Supporting Fig. S4:** (a, b) Two different magnifications (50, 10 nm) of TEM and (c) HR‐TEM images of CAZ (d) Selected area of mapping and corresponding EDX mapping images of the CAZ NPs. **Supporting Fig. S5:** (a, b) Two different magnifications (50, 10 nm) of TEM and (c) HR‐TEM images of (CAZ/Gr)S (d) Selected area of mapping and corresponding EDX mapping images of the (CAZ/Gr)S NCs. **Supporting Fig. S6:** N2 adsorption‐desorption isotherms of (a) CAZ, (b) (CAZ/Gr)S, and (c) (CAZ/Gr)H, (d) The pore size distribution of CAZ, (CAZ/Gr)S and (CAZ/Gr)H NCs. **Supporting Fig. S7:** XPS pattern of CAZ (a) Zn 2p, (b) Cu 2p, (c) Ag 3d, and (d) O 1s. **Supporting Fig. S8:** (a) UV‐Vis spectrum and (b) Tauc plot of CAZ, (CAZ/Gr)S and (CAZ/Gr)H.

## Conflicts of Interest

The authors declare no conflicts of interest.

## Supporting information

Supplementary Material
