# Supplementary material for: Comparative Study of Hydrothermal and Sonochemical Preparation of Cu and Ag Codoped ZnO/Graphene Nanocomposites for Enhanced Catalytic and Inactivation of Pathogens
Source: Chemphyschem. 2026 Feb 15;27(3):e202500256. doi: 10.1002/cphc.202500256 (PMC12906927; doi:10.1002/cphc.202500256)
Supplement: Supplementary file 1 — Supplementary Material [file CPHC-27-e202500256-s001.pdf]

Supporting Information

Comparative Study of Hydrothermal and Sonochemical Preparation of Cu and Ag co-doped ZnO/Graphene Nanocomposites for Enhanced Catalytic and Inactivation of Pathogens by Using Visible light

Jarvin Mariadhas[a], Vinodhkumar Ganesan[b], Sudhan Narayanan[c], Sarojini Jeeva Panchu[d]*, Hendrik C. Swart*[d] Manueldoss Beaula Ruby Kamalam[a], Nelson Y. Dzade[e] Savariroyan Stephen Rajkumar Inbanathan[a]*.

[a] Post Graduate and Research Department of Physics, The American College, Madurai, PIN: 625002, Tamil Nadu, India.

[b] Post Graduate and Research Department of Physics, Raja Doraisingam Government Arts College, Sivagangai, PIN: 630561, Tamil Nadu, India.

[c] Post Graduate and Research Department of Chemistry, Thiagarajar College, Madurai, PIN: 625009, Tamil Nadu, India.

[d] Department of Physics, University of the Free State, PO Box 339, Bloemfontein, 9300, South Africa.

[e] Department of Energy and Mineral Engineering, Pennsylvania State University, University Park, State College, PA 16802, United States.

corresponding author:

stephenrajkumarinbanathan@americancollege.edu.in (Savariroyan Stephen Rajkumar Inbanathan)

Panchu.SJ@ufs.ac.za (Sarojini Jeeva Panchu)

SwartHC@ufs.ac.za (Hendrik C Swart)

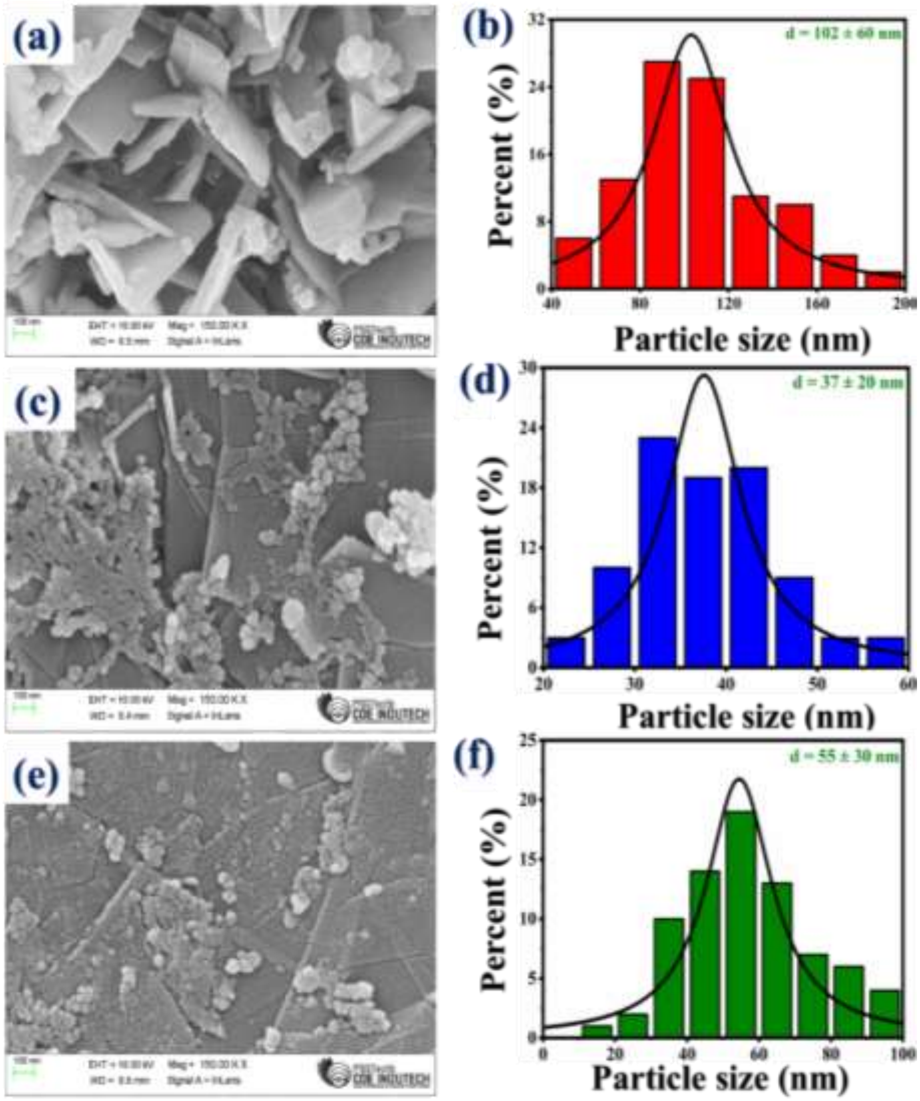

Fig.S1: FESEM image of (a) CAZ NPs, (c) $(CAZ/Gr)_{S,}$ and (e)$(CAZ/Gr)_{H}$, and the size distribution of the (b) CAZ NPs, (d) $(CAZ/Gr)_{S,}$ and (f)$(CAZ/Gr)_{H}$ based on the FESEM image.

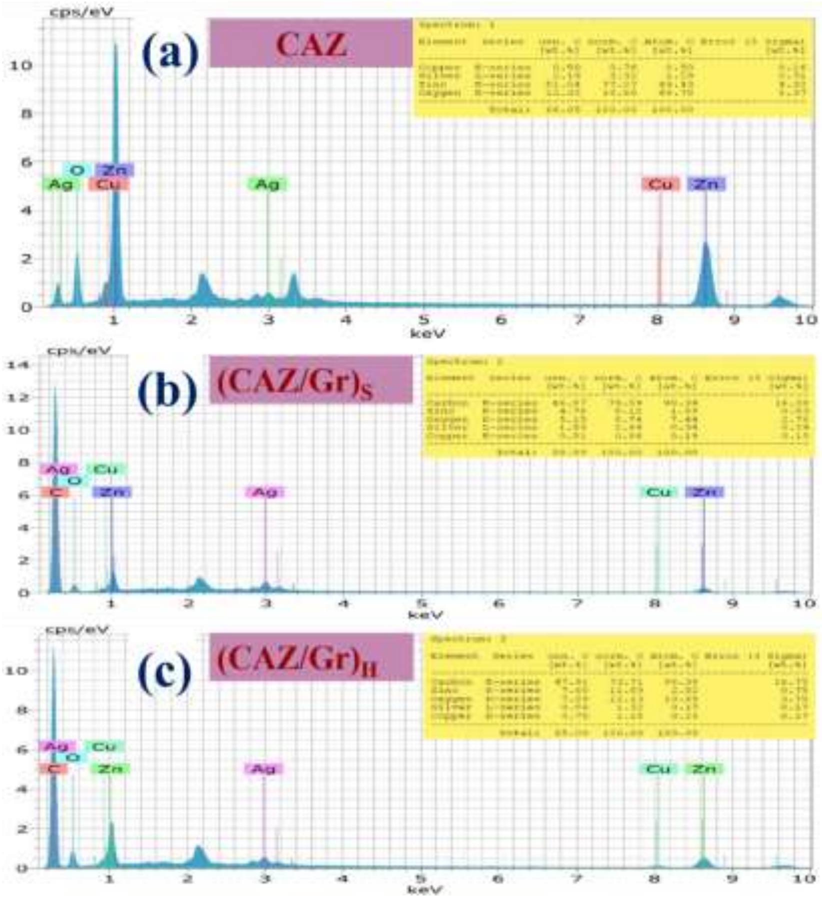

Fig. S2: EDX images of (a) CAZ NPs, (c) $(CAZ/Gr)_S$, and (e) $(CAZ/Gr)_H$.

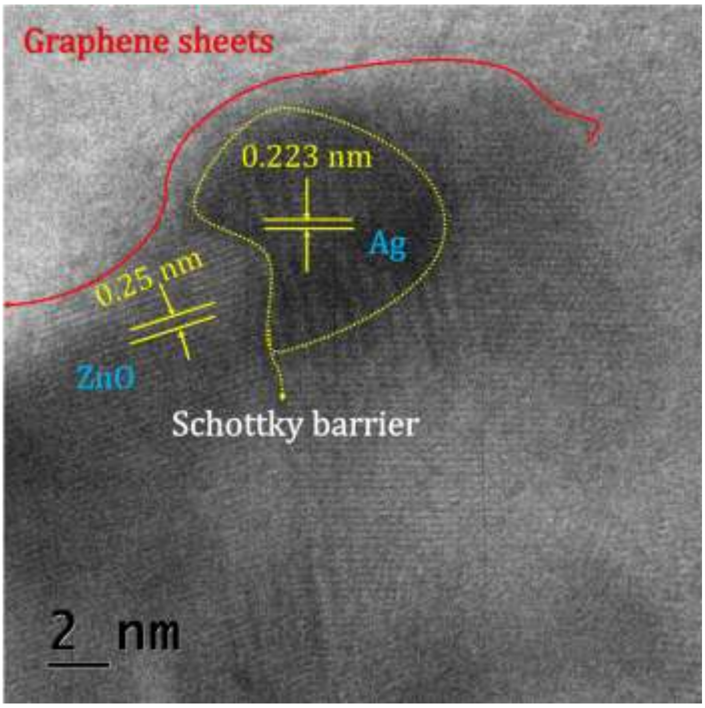

Fig. S3: Schottky potential barrier in $(CAZ/Gr)_H$ NCs.

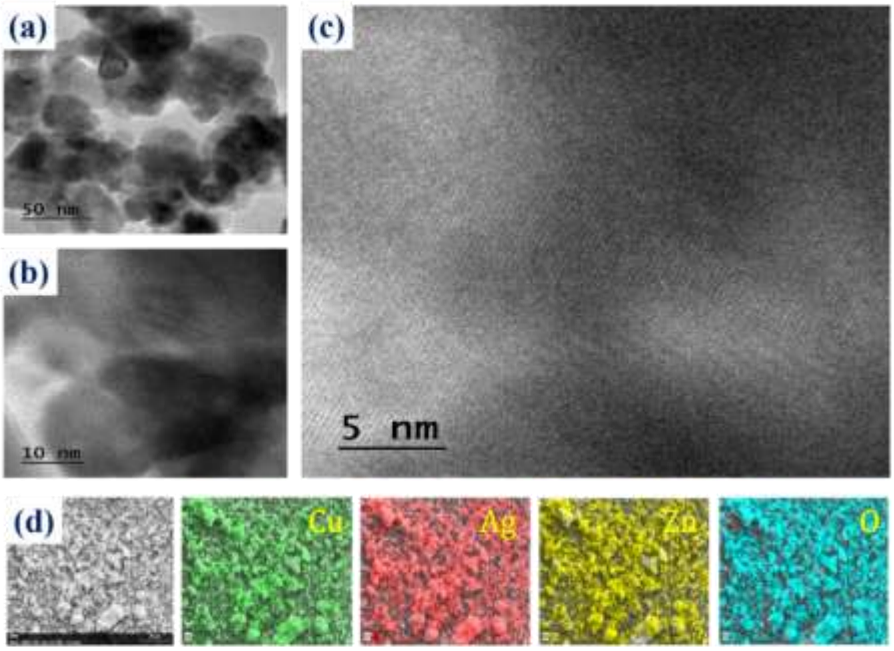

Fig. S4: (a, b) Two different magnifications (50, 10 nm) of TEM and (c) HR-TEM images of CAZ (d) Selected area of mapping and corresponding EDX mapping images of the CAZ NPs.

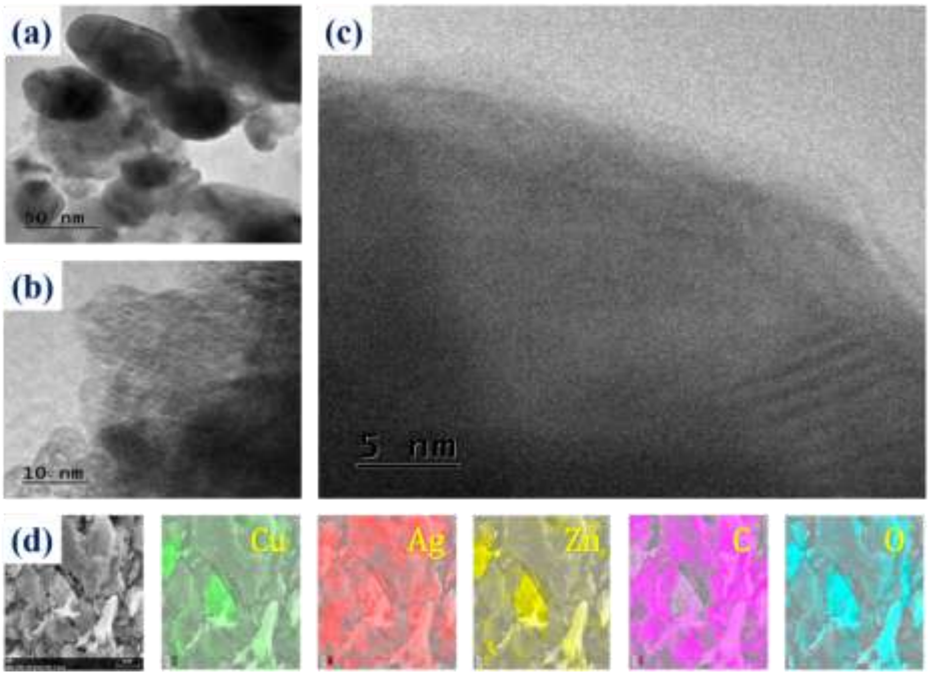

Fig. S5: (a, b) Two different magnifications (50, 10 nm) of TEM and (c) HR-TEM images of $(CAZ/Gr)_S$ (d) Selected area of mapping and corresponding EDX mapping images of the $(CAZ/Gr)_S$ NCs.

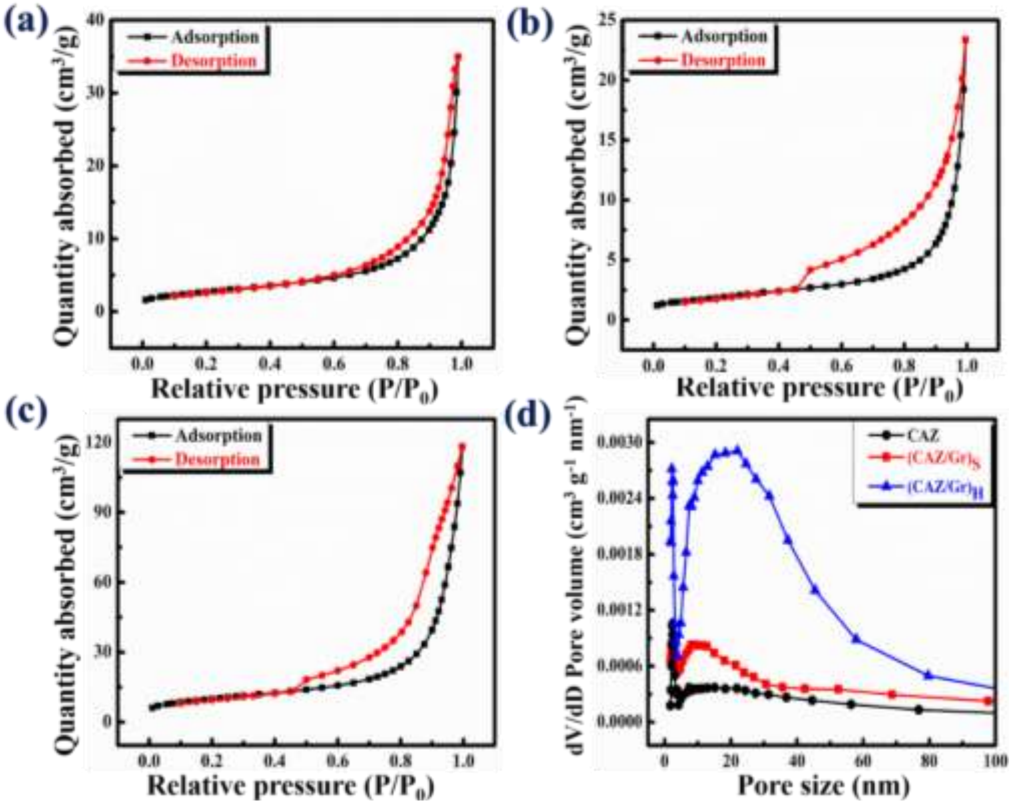

Fig. S6: N_2 adsorption-desorption isotherms of (a) CAZ, (b) $(CAZ/Gr)_S$, and (c) $(CAZ/Gr)_H$, (d) The pore size distribution of CAZ, $(CAZ/Gr)_S$ and $(CAZ/Gr)_H$ NCs.

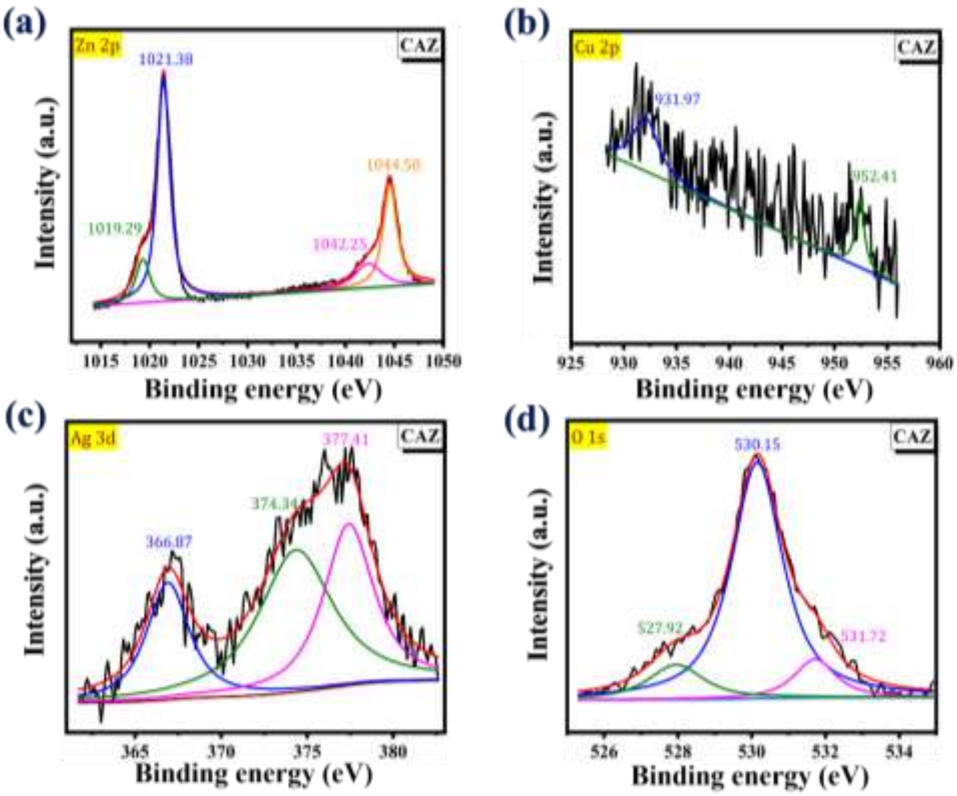

Fig. S7: XPS pattern of CAZ (a) Zn 2p, (b) Cu 2p, (c) Ag 3d, and (d) O 1s

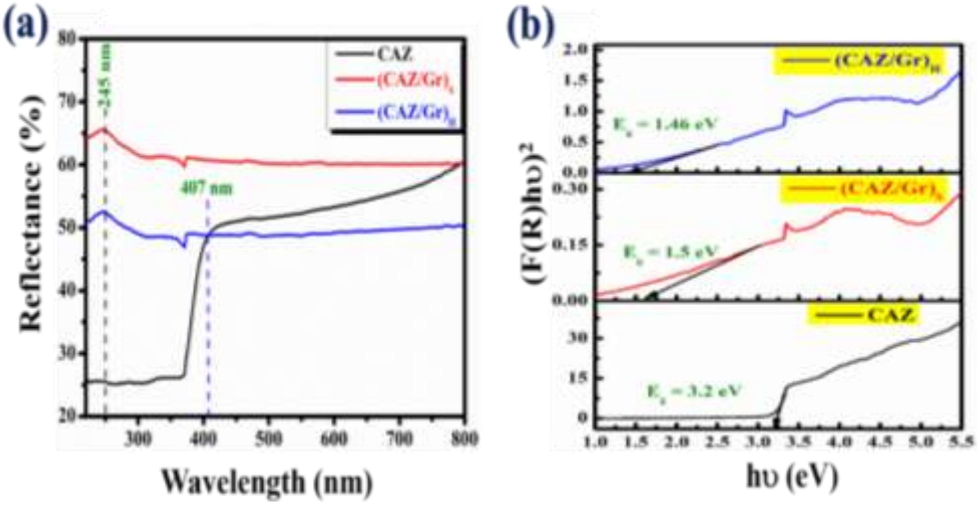

Fig. S8: (a) UV-Vis spectrum and (b) Tauc plot of CAZ, $(CAZ/Gr)_S$ and $(CAZ/Gr)_{H.}$
